# Importance of disulphide bonds for vaccinia virus L1R protein function

**DOI:** 10.1186/1743-422X-2-91

**Published:** 2005-12-09

**Authors:** Robert E Blouch, Chelsea M Byrd, Dennis E Hruby

**Affiliations:** 1 Department of Microbiology, Oregon State University, 220 Nash Hall, Corvallis, Oregon, 97331, USA; 2 Siga Technologies, 4575 SW Research Way, Suite 230, Corvallis, Oregon, 97333, USA

## Abstract

L1R, a myristylated late gene product of vaccinia virus, is essential for formation of infectious intracellular mature virions (IMV). In its absence, only viral particles arrested at an immature stage are detected and no infectious progeny virus is produced. Previous studies have shown that the L1R protein is exclusively associated with the IMV membrane and that myristylation is required for correct targeting. The L1R protein contains six cysteine amino acid residues that have all been shown to participate in intramolecular disulphide bonds. However, it was not clear what role, if any, the disulfide bonds play in the membrane topology of the L1R protein. To address this question, a comprehensive library of L1R mutants in which the cysteine residues have been mutated to serine (either individually or in combination) were tested for their ability to rescue a L1R conditional lethal mutant virus under non-permissive conditions. Much to our surprise, we determined that C57 was not essential for production of infectious IMV. These results suggest that protein disulphide isomerases may be involved in reorganization of disulfide bonds within the L1R protein.

## Findings

Vaccinia virus (VV) continues to be the model organism for the investigation of the Orthopoxviridae family and as a result is the most widely studied and best understood virus in this family. This being said, our understanding of this virus family is still limited due to the size and complexity of these DNA viruses which maintain a broad host range having members that infect insects (entomopoxviruses) and a large number of vertebrates (chordopoxviruses). Two poxviruses known to cause disease in human hosts are variola, the causative agent of smallpox and Molluscum contagiosum, which causes small tumors on the skin and is an opportunistic pathogen in AIDS patients. Largest of the DNA viruses, the poxvirus genome encodes more than 200 gene products. One reason for the sheer number of genes is the viruses' unique ability to replicate its genome, form complex macromolecular structures and assemble infectious viral particles solely within the cytoplasmic compartment of infected cells.

It has previously been shown that the product of the VV L1R open reading frame is essential for the formation of intracellular mature virions (IMV) and plays a role in virion morphogenesis [1-4]. In the absence of L1R, only immature virion particles are formed and proteolytic cleavage of core proteins does not occur [1]. This prevents core condensation and arrests virion morphogenesis at a non-infectious stage. L1R is the target of neutralizing antibodies to IMV [5], therefore making it a potential target for the development of antivirals. However, the biological function of L1R remains largely unknown. L1R contains six conserved cysteine residues that have been shown to be oxidized to form three intramolecular disulphide bonds [6]. These are believed to be essential for correct protein folding and proper function. In addition, they may serve as a membrane attachment factor, playing a role in trafficking of L1R to the endoplasmic reticulum-golgi intermediate compartment (ERGIC).

In this report conditional-lethal expression of L1R and complementation with a library of cysteine-to-serine L1R mutants was used to investigate the importance of disulphide bond formation and the presence of the contributing cysteine residues to protein function.

A recombinant virus was constructed in which the expression of the L1R gene could be regulated by the presence or absence of TET using the components of the bacterial tetracycline operon [7]. This system has previously been shown to be successful in the regulation of the vaccinia virus I7L [8], G1L [9,10] and A14L [11] genes. A plasmid containing the tetracycline operator (TetO) just upstream of the L1R open reading frame (ORF) and including flanking genomic DNA sequence (including the native promoter) to aid in homologous recombination was used to create the recombinant virus vvTetO:L1R. T-Rex-293 cells (Invitrogen) which express the tetracycline repressor (TetR) were used to regulate expression of the L1R gene from the inducible mutant virus.

**Figure 1:**
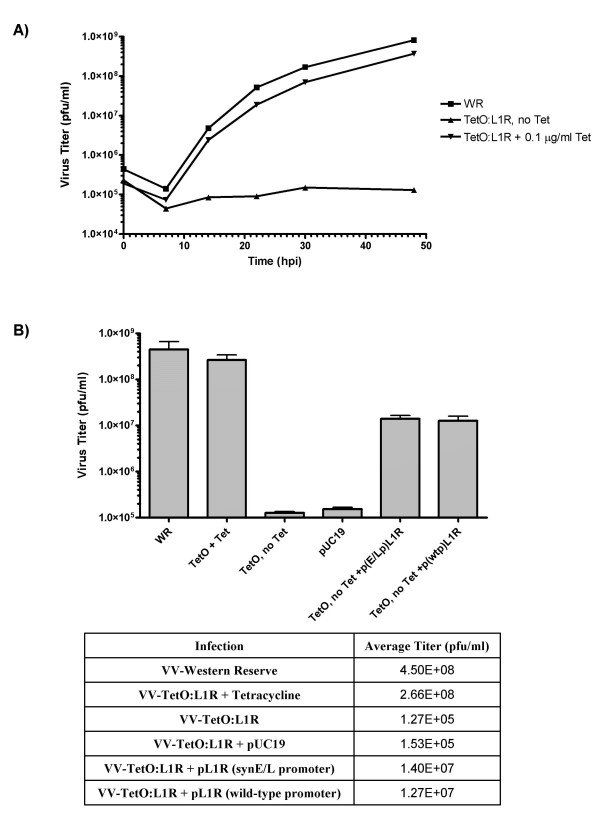
(**A**) Growth-curve kinetics comparing vv:Western Reserve to vv:TetO:L1R under permissive and non-permissive conditions. Each infection was performed at an MOI of 0.1 pfu and harvested at various times from 0 to 48 hpi and the resulting cell lysates were titered using BSC_40 _cells. (**B**) Transiently expressed L1R is capable of phenotypic rescue of conditional-lethal viral infection under non-permissive conditions. Infections were performed at 0.1 MOI with either VV-WR (WR) or VV-TetO:L1R (TetO) in the absence of tetracycline unless noted. Transfections of plasmid DNA were performed using 2 μg of pUC19, p(E/Lp)L1R or p(wtp)L1R. All infections were harvested at 24 hpi and titered on BSC_40 _cells.

To verify that expression of L1R is essential for viral replication and can be regulated by tetracycline (Tet), a growth curve in the presence and absence of Tet was performed (Figure 1A). Under permissive conditions, in the presence of 0.1 μg/ml Tet, vvTetO:L1R grew to the same yield and with the same kinetics as wild type virus. However, in the absence of Tet, there was over a 3-log decrease in viral titer. Transfection of plasmid borne L1R, driven off of either its native promoter (p(wtp)L1R or a synthethic early/late promoter (p(E/Lp)L1R), resulted in a greater than 100-fold increase in infectious progeny virus over the control with no transfected DNA. (Figure 1B). There was concern that L1R being expressed constitutively at all times during infection as opposed to only at late times might negatively impact viral yield or in some way interrupt or slow the viral life cycle. This did not occur, most likely because three proteins, essential for disulphide bond formation in L1R, are expressed as late proteins. Without G4L, A2.5L, and E10R present early in the infection, L1R was not in its active conformation. Its presence in non-disulphide bonded form does not appear to hinder virion morphogenesis or viral assembly.

**Figure 2:**
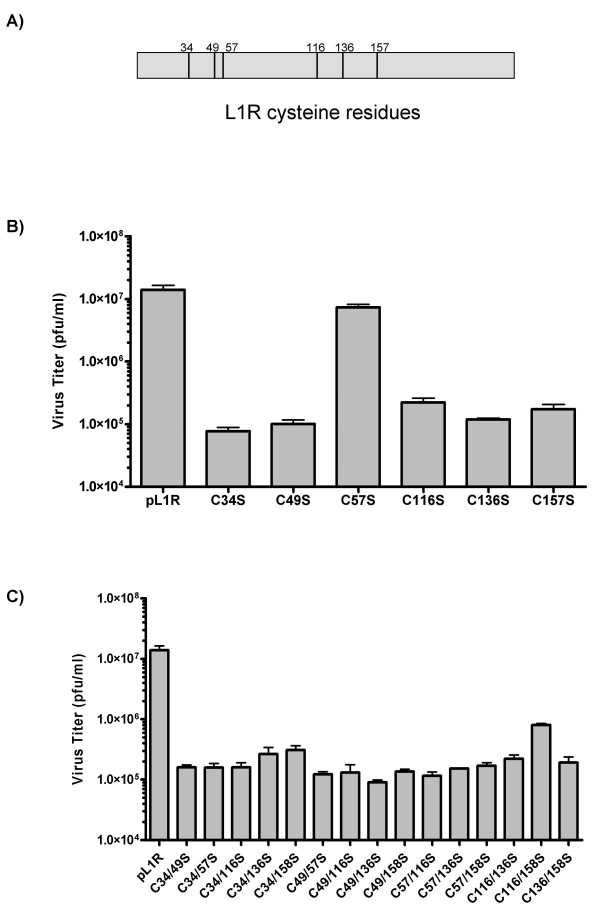
(**A**) Diagram of the location of the six cysteine residues in L1R. (**B**) Transient Expression of L1R cysteine-to-serine single mutants. Infections were performed by transfection of 2 μg of pL1R each containing a single Cysteine-to-Serine mutation in the coding sequence at time of infection with VV-TetO:L1R at an MOI of 0.1 under non-permissive conditions. Infections were harvested at 24 hpi and titered on BSC_40 _cells. (**C**) Transient Expression of L1R cysteine-to-serine double mutants. Infections were performed by transfection of 2 μg of pL1R containing double Cysteine-to-Serine mutations in the coding sequence at time of infection with VV-TetO:L1R at an MOI of 0.1 under non-permissive conditions. Infections were harvested at 24 hpi and titered on BSC_40 _cells.

L1R contains six cysteine amino acids that bind through disulphide bridges to form three stable intramolecular bonds in the active form of the protein [12]. Figure 2A shows the location of the six cysteine residues involved in disulphide bonding. In order to determine if all three bonds are essential to protein function and elucidate possible partnering models, plasmid DNA containing the L1R ORF expressed from the synthetic early/late promoter and containing individual cysteine to serine mutants were expressed during infections with vvTetO:L1R under non-permissive conditions. Five of the six mutants (C34S, C49S, C116S, C136S, and C158S) were incapable of rescuing the infections. Interestingly, L1R lacking the third cysteine at amino acid 57 was capable of 52% rescue and suggests that participation of C57 appears to be non-essential for protein function (Figure 2B). Rescue experiments were also performed using double mutants of L1R containing every possible variation of two cysteine-to-serine tandem mutants. The results showed that none of the double mutants were capable of significant rescue (Figure 2C).

The Tet operon conditional-lethal system has been used to study the effects of mutations introduced into L1R and transiently expressed during infections under non-permissive conditions. Utilizing this approach, it was shown that five of the six cysteine amino acids present in wild-type L1R are essential for proper L1R function and active conformation. The single cysteine at aa-57 was shown to be non-essential in this role. This presents a puzzle considering there is previous research suggesting that there are three intramolecular disulphide bonds utilizing all six cysteine residues [12]. Confocal microscopy comparing wild-type infections and infections with transiently expressed mutant L1R verified that the protein was being made in from all six constructs (data not shown). It appeared that trafficking of L1R to the proper membrane may be dependent upon proper disulphide bonding as localization was altered in Cys-49 and Cys 116 mutants. These findings suggest the possibility that a cellular or virally encoded protein disulphide isomerase is required for proper disulphide pairing in active L1R. It is conceivable that cyteine-57 forms an incorrect disulphide pairing as an intermediate. Protein disulphide isomerase is then necessary to resolve this mispairing and the disulphide bond that is formed by Cys-57 and its unknown partner is not necessary for functional L1R. This is further evidenced by the crystal structure of L1R [12]. The terminal protein in the disulphide forming redox pathway is G4L [6], a cytoplasmic protein. If disulphide bonding of L1R were to occur in this fashion in the cytoplasm, the trafficking effects of the N-terminal myristoyl group, which would be hidden within L1R, could be lost. This could be circumvented if G4L established an intermediate disulphide bond configuration that exposes the myristoyl group and allows trafficking to the ERGIC. Then, once incorporated into the membrane of the ERGIC, the bonds are isomerized, converting L1R to its active confirmation. The ERGIC associated A2.5L/E10R heterodimer contains two C-XXX-C motifs that have been established in DsbC homodimers in *E. coli *[13]. The C57S mutant was capable of better than 50% rescue of infection under non-permissive conditions when compared to rescue with wild-type L1R. This small decrease could be attributed to lack of the third bond, however its absence does not abolish function.

A series of tandem mutants utilizing every combination of two cysteine-to-serine mutations was also tested in the same manner. None of the mutants were capable of significant rescue under non-permissive conditions. This is not surprising based upon observation of the single mutants except in one respect: it suggests that if there is a second incorrect pairing that a protein disulphide isomerase is needed to correct, that this is an essential intermediate and without it the correct bond alignment cannot be achieved. The virally encoded redox pathway described by Senkevich *et al *(2002), contains disulphide linked E10R and A2.5L which they compared to *E. coli *DsbB and yeast ERO1p and ERV2p which contain two pairs of active cysteine residues. G4L is likened to the downstream thioredoxin-like proteins DsbA in *E. coli*, and PDI and its homologues in the ER of yeast. It is possible that isomerase activity during vaccinia infection is achieved by one of the known viral redox proteins or by another, yet unknown viral protein. This activity, if shown to exist is not likely to be attributed to a cellular protein, as disulphide bond formation and isomerase activity is believed to occur solely in the lumen of the ER.

This study has shown that only two of the three intramolecular disulphide bonds are essential for L1R to perform its function in formation of infectious IMV particles. Cysteine residues at positions 34, 49, 116, 136 and 158 are essential for protein function and viral propagation. The cysteine at position 57 is non-essential and its partnering capabilities are not necessary for proper function of L1R. Cys-49 and Cys-116 disrupted localization if L1R as evidenced by confocal microscopy. The results also suggest the presence of isomerase activity in L1R bond reshuffling and that it may be a required factor in promoting proper protein conformation and function. Here, we propose that an incorrectly disulphide bonded intermediate mediates trafficking of L1R to the membrane of the ERGIC, where isomerization of these bonds results in an active conformation with the myristoyl group hidden with the hydrophobic cavity of the active protein.

## Competing interests

The author(s) declare that they have no competing interests.

## Authors' contributions

CMB constructed the recombinant virus. REB conducted the experiments. CMB and REB wrote the manuscript. DEH conceived the study, coordinated the research efforts and edited the paper. All authors read and approved the final manuscript.
